# Renal Vein Blood Flow Patterns Identify Patients at Risk for Early Kidney Allograft Loss Due to Cardiac Postrenal Vein Congestion

**DOI:** 10.3390/jcm14144897

**Published:** 2025-07-10

**Authors:** Franz Josef Putz, Paul Christian Kranert, Miriam C. Banas, Wilma Schierling, Ernst Michael Jung, Tobias Bergler, Bernhard Banas

**Affiliations:** 1 Department of Nephrology, University Hospital Regensburg, 93053 Regensburg, Germany; 2 Department of Vascular Surgery, University Hospital Regensburg, 93053 Regensburg, Germany; 3 Department of Radiology, University Hospital Regensburg, 93053 Regensburg, Germany; 4 Department of Nephrology, Hospital Ingolstadt, 85049 Ingolstadt, Germany

**Keywords:** kidney transplantation, cardiorenal syndrome, congestive heart failure, delayed graft function, venous visceral congestion, ultrasound, duplex sonography

## Abstract

**Background/Objectives**: Early graft loss within the first year is a rare complication of renal transplantations. In some cases, venous congestion may cause renal dysfunction, but, so far, this syndrome has been assessed by the presence of the triad of an unexplained decrease in renal function together with severe volume overload, relevant heart disease, and a typical histopathological pattern of tubular injury. This study aimed to determine the proportion of patients with early allograft loss due to venous congestion within the first year after transplantation. Additionally, we characterized typical renal vein flow profiles to identify patients at risk of early graft loss due to postrenal venous congestion and prerenal perfusion deficit. **Methods**: In this retrospective, single-center study, patients who underwent kidney transplantations between 2010 and 2020 and experienced early graft loss within the first year after transplantation were included. Clinical data and renal vein blood flow profiles were collected retrospectively. **Results**: A total of 579 patients received kidney transplants between 2010 and 2020. Of these, 43 patients (7.4%) lost their grafts within the first year of transplantation. Nine of these 43 patients (20.9% with early graft loss) lost their graft due to a suspected cardiorenal syndrome. Besides graft loss, cardiorenal patients had a significantly higher risk of death than other patients. All cardiorenal patients could be identified using a distinct renal vein blood flow profile (100%). **Conclusions**: We characterized the typical renal vein blood flow profiles in patients at risk of premature graft loss due to venous congestion. The early identification of such patients is crucial in improving outcomes after renal transplantation.

## 1. Introduction

Kidney transplantation is the treatment of choice for patients with end-stage renal disease, in addition to various dialysis procedures [1]. Compared to dialysis, patients benefit from better quality of life, a higher functional level, and longer survival after successful kidney transplantation [2,3].

Early graft loss within the first year after renal transplantation occurs in less than 5% of all deceased renal transplant patients [4]. The main reasons for early graft loss are surgical problems, early immunological reasons, and death with a functioning graft. In some cases, the reason for early graft loss remains unclear.

In recent years, insights have been obtained into the vital interaction between cardiac function and the kidney, known as the cardiorenal axis, which, if impaired, causes different types of cardiorenal syndromes [5]. Few studies have addressed the consequences of cardiorenal syndrome in renal transplant patients—for example, in relation to graft loss. These studies describe patients as having the triad of an unexplained decrease in renal function together with severe volume overload, relevant heart disease, and a typical histopathological pattern of tubular injury [6,7,8]. To date, these patients have only been classified by clinical assessment.

Cardiorenal syndrome consists of several components, of which two problems predominate hemodynamically: prerenal perfusion deficit and postrenal venous congestion. These aspects can be present both independently of each other and together in different forms.

Recently, more information has been acquired regarding the concept of venous congestion within the kidney as a consequence of acute or chronic right-sided heart failure [9]. The renal vein Doppler profile has been recognized as a possible marker describing the degree of renal congestion [10,11]. There is evidence that an impaired renal vein Doppler profile can help to predict the likelihood of adverse renal events in patients after cardiac surgery [12], intensive care patients [13], or patients with heart failure [14]. In other patient groups (e.g., pulmonary hypertension [15] and cardiac failure [16,17]), patients with a higher degree of venous congestion have inferior outcomes and a higher risk of death than patients without pathological findings on renal vein Doppler sonography. To date, no study has described the extent of venous congestion in renal transplants based on the Doppler profile of the renal veins.

The question arises of whether a relevant proportion of graft failures within the first year is provoked by postrenal venous congestion and prerenal perfusion deficit. In this retrospective study, we analyzed the reasons for early graft loss in patients within the first year after renal transplantation at our transplant center between 2010 and 2020. In the second step, we identified the percentage of patients with assumed cardiorenal syndrome. In the third step, we retrospectively characterized the differences in the renal vein Doppler profiles and other hemodynamic parameters of the identified cardiorenal patients in comparison to patients with other reasons for premature graft loss.

## 2. Materials and Methods

This retrospective study included all patients who received a renal transplant between the years 2010 and 2020 at the University Hospital Regensburg (Germany). Patient data were retrieved from a clinical database with permission from the local ethics committee (17-662-101_P1).

Patients with early graft loss within the first year after transplantation were identified and categorized according to the following reasons for graft loss: surgical, immunological, poor organ quality, thromboembolic events, cardiorenal syndrome, death with a functioning graft, death without a functioning graft, and unknown reasons.

**Figure 1:**
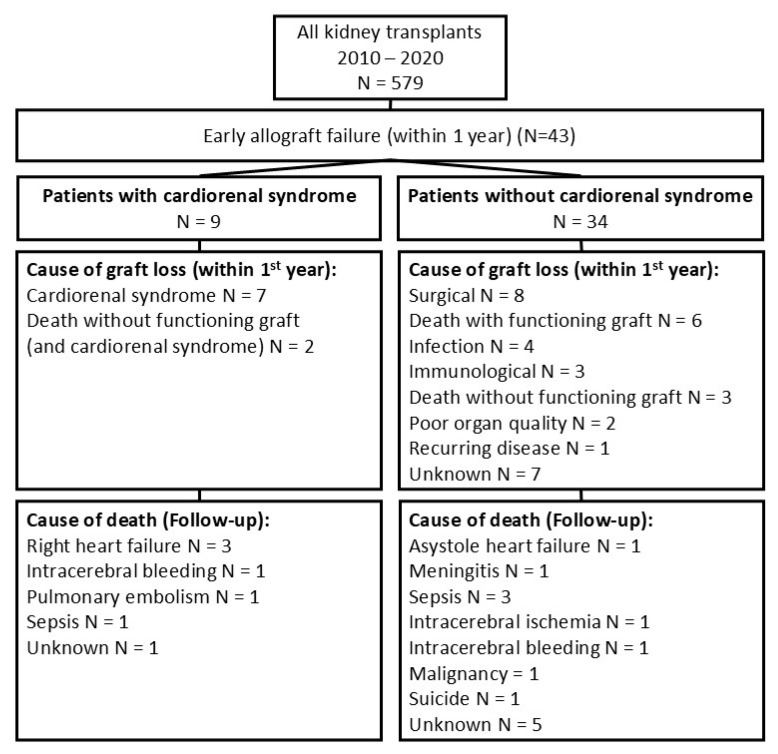
Flow chart.

Cardiorenal syndrome was assumed if patients fulfilled the following criteria: (i) insufficient renal function (defined as impaired renal function of the renal transplant, e.g., eGFR < 15 mL/min/1.73 m^2^, and/or the necessity of the continuation of dialysis after transplantation) without any other obvious reasons, as proven by biopsy; (ii) severe volume overload (>5 kg over the post-dialysis dry weight or the necessity of dialysis due to volume overload); and (iii) relevant heart disease. Cardiorenal syndrome was diagnosed after the interdisciplinary discussion of the case with nephrologists, surgeons, and cardiologists. Renal biopsy was performed in all patients to rule out other reasons for graft loss (e.g., rejection). Only one of the cardiorenal patients was without a renal biopsy (patient 3). In patients with suspected cardiorenal syndrome, acute tubular injury was the main finding in the histological analysis. In some cases, the detected acute tubular injury was accompanied by minimal additional findings (e.g., borderline rejection and UTI) in the biopsy. The reasons for death were recorded and categorized (e.g., cardiovascular, heart failure, intracerebral bleeding, sepsis, suicide) (Figure 1).

Post-transplant treatment was performed according to in-house standards. Standard immunosuppression was based on a calcineurin inhibitor (tacrolimus), antimetabolites (mycophenolic acid), and steroids. Induction therapy was based on basiliximab or thymoglobulin, according to the patient’s individual immunological risk.

**Figure 2:**
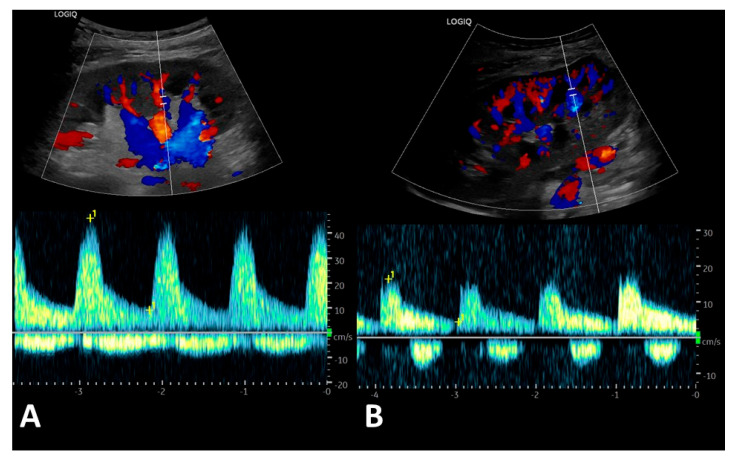
Sonographic assessment of the transplant renal vein flow profile. Color-coded duplex sonography of renal transplantation. The renal blood flow of the renal artery and vein was recorded within the blood vessels at the parenchyma–pyelon boundary (Aa. and Vv. interlobares). Arterial signals highlight the cardiac cycle. (**A**) Typical continuous renal vein flow profile in a patient without cardiorenal syndrome. (**B**) Discontinuous renal vein flow profile in a patient with cardiorenal syndrome.

Based on the medical records of all patients, the ultrasound results and Doppler spectra were retrospectively reviewed by an experienced nephrologist and sonographer (Figure 2). In all patients, the renal vein flow profile was analyzed based on the original image material retrieved from the patient’s data system. Doppler spectra were measured at the border of the parenthesis to the pyelon. Different ultrasound devices were used in the present study.

**Figure 3:**
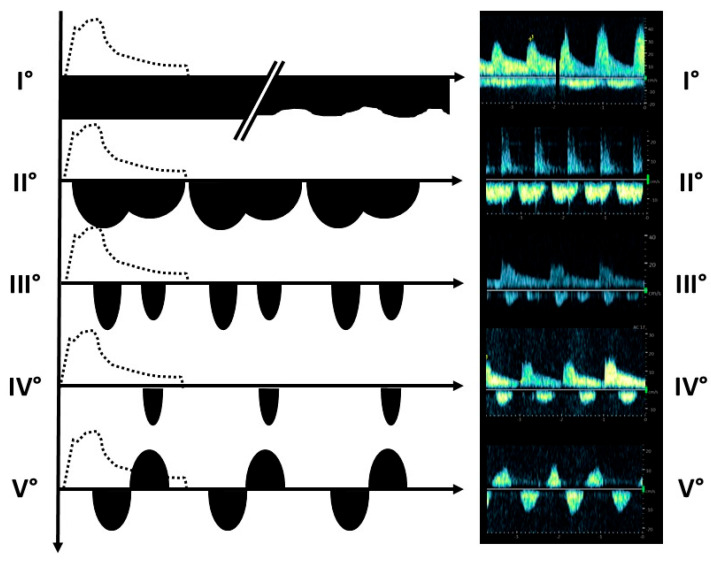
Classification of the renal vein blood flow pattern. Venous blood flow was categorized according to different renal vein blood flow patterns. The idealized arterial flow signals are indicated by dotted lines. The venous blood flow was continuous linear or continuous undulant (Grade I), continuous biphasic (Grade II), discontinuous biphasic (Grade III), discontinuous monophasic (Grade IV), or to-and-fro motion (Grade V) in the renal vein. An increasing grade within this classification was associated with increased venous congestion. © Putz, all rights reserved.

Based on our previous experience and the existing literature [11,15,18], we adapted and developed a grading system to define venous blood flow profiles in renal transplants. As shown in Figure 3, we categorized venous blood flow into continuous linear or undulant (Grade I), (nearly) continuous biphasic (Grade II), discontinuous biphasic (Grade III), discontinuous monophasic (Grade IV), or to-and-fro motion (Grade V) in the renal vein. In contrast to the other grading systems (e.g., the VExUS score), we added Grade V because we found this distinct renal vein blood flow type in patients with massive venous congestion. We assumed that this grading system could be used to estimate the degree of venous congestion. According to our clinical experience, Grade I was a normal renal vein flow pattern, and Grade II could be a transitional stage, whereas Grades III–V were predominant in patients with severe postrenal venous congestion.

Laboratory tests, renal biopsies, and other instrumental tests concerning hemodynamics (e.g., abdominal ultrasound, echocardiography, coronary angiography, and right heart catheterization) were included in the analysis. Acute transplant rejection was diagnosed based on the current version of the BANFF classification.

Patient records were also screened retrospectively for signs of acute or chronic right ventricular failure before renal transplantation (e.g., abdominal ultrasound or echocardiography). For the assessment of liver duplex sonography, Doppler spectra of the liver vein, portal vein, and hepatic artery were evaluated.

Survival rates were analyzed using Kaplan–Meier plots. Significant differences were indicated using Student’s *t*-test (*p* < 0.05), Pearsons’ chi^2^ test, and the log-rank test. In this case, statistical analyses were performed using SPSS (IBM SPSS Statistics Version 29.0.0.0, Armonk, NY, USA).

## 3. Results

### 3.1. Patient Characteristics and Clinical Outcomes

Between 2010 and 2020, 579 patients received a kidney transplant at the University Hospital of Regensburg (Germany). In total, 425 patients received an organ from a deceased donor (73.4%), and 154 patients received transplants from living donors (26.6%).

We identified 43 patients (7.4%) with an early transplant failure within the first year after transplantation (including death with a functioning graft). This reflects a 1-year graft survival rate of 92.6%. Fifteen patients died within the first year, which reflects a 1-year patient survival rate of 97.4%. 

The overall reasons for early graft loss were surgical (n = 8, 18.6%), immunological (n = 4, 9.3%), poor organ quality (n = 2, 4.7%), death with a functioning graft (n = 6, 14.0%), infection (n = 4, 9.3%), death without a functioning graft and no cardiorenal syndrome (n = 3, 6.9%), and unknown reasons (n = 7, 16.2%). Cardiorenal syndrome was suspected in nine patients (20.9%), including two patients who died without a functioning graft.

Fifteen patients died within the first year, and 21 patients died during the follow-up. Six patients died with a functioning graft, and five patients died without a functioning graft. The causes of death within the first year were sepsis (n = 3), cardiac death (n = 1), right heart failure (n = 2), intracranial bleeding (n = 2), intracerebral infarction (n = 1), meningitis (n = 1), pulmonary embolism (n = 1), suicide (n = 1), and unknown (n = 3). Six patients died during the follow-up period, after the first year. The reasons were unknown (n = 3), intracerebral infarction (n = 1), malignancy (n = 1), and right-sided heart failure (n = 1).

### 3.2. Identifying Patients with Graft Loss Due to Cardiorenal Syndrome

After identifying the reasons for graft loss, the patients were divided into two groups: patients with and without suspicion of cardiorenal syndrome (Figure 1). These groups were analyzed based on the patients’ characteristics and reasons for graft failure. The recipient criteria, donor criteria, and surgical or immunological parameters did not differ significantly between the groups (Table 1).

**Figure 4:**
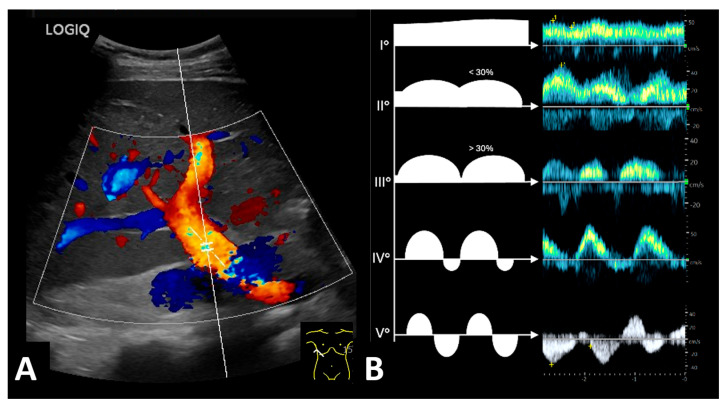
Different blood flow patterns of the liver portal vein in patients with increasing stages of venous congestion. (**A**) Color-coded duplex sonography of the portal vein. (**B**) Blood flow patterns. Grade I: Typical undulant hepatopetal blood flow in a patient without venous congestion. Grade II and III: Increased pulsatility of the portal vein (variability < 30% vs. >30%). Grade IV: Discontinuous blood flow with an end-diastolic backflow. Grade V: To-and-fro blood flow as the maximal grade of venous congestion in the liver. © Putz, all rights reserved.

The quality of the echocardiography data prior to kidney transplantation was limited, and information was missing for one patient in the cardiorenal group. While the statistical significance should be interpreted with caution due to the limited data quality, it can be concluded that patients with graft loss due to cardiorenal syndrome have significantly more cardiac comorbidities and exhibit significantly greater pathological echocardiography parameters. Based on our data, there was a relevant proportion of patients with singular right heart problems. Patients with cardiorenal syndrome were significantly more likely to have evidence of venous congestion on liver duplex sonography before transplantation (Table 2, Figure 4).

### 3.3. Post-Transplant Doppler Sonography Parameters of Transplanted Kidneys

The post-transplant duplex parameters of all patients were reviewed (Figure 2). Five types of renal vein profiles were identified and graded (Figure 3). High grades of venous blood flow indicate increasing cardiac postrenal venous congestion; however, only patients with ongoing cardiorenal syndrome showed higher grades of renal venous flow (Grades IV and V). All patients without clinical signs of cardiorenal syndrome had low grades with reference to the venous flow profiles (Grades I–II) (Table 3). Grade III was not found in the patients in this study, but we have noticed this renal vein blood flow profile in other renal transplant patients with impending cardiorenal syndrome outside of this study.

Table 4 shows the clinical and sonographic characteristics of patients with cardiorenal syndrome. All cardiorenal patients were identified by analyzing the renal vein blood flow profile.

### 3.4. Pre-Transplant Liver Doppler Sonography Identifies Patients at Risk for Cardiorenal Syndrome

Retrospectively, cardiorenal patients had more severe heart disease (Table 2) but also showed a higher rate of venous congestion in liver duplex sonography. We were able to identify 75% of the cardiorenal patients retrospectively by the identification of an alternative blood flow spectrum in the routine liver duplex sonography before the renal transplantation.

These patients had a higher rate of venous congestion in the liver. The sonographic assessment of the volume status of dialysis patients mostly includes dilatation of the inferior vena cava. However, signs of relevant venous congestion could be identified in the liver and portal veins. We found to-and-fro motion within the liver vein as the first sign of relevant venous congestion of the liver. However, in our experience, cardiorenal patients typically show an alternative blood flow profile within the portal vein; instead of the typical undulant hepatopetal blood flow (Grade I), patients with increasing signs of venous congestion first show increased pulsatility (Grades II and III) or a discontinuous blood flow with an end-diastolic backflow (Grade IV) in the portal vein. In the maximal form of venous congestion, a to-and-fro blood flow was observed within the portal vein (Grade V). These alternative portal vein flow profiles could be found in 75% of the patients with graft loss due to cardiorenal syndrome before renal transplantation.

All other patients with early graft failure due to other reasons had these signs in only a small proportion (7.2%). Interestingly, alternative liver flow profiles could be also found in most patients with manifest cardiorenal syndrome after transplantation (75.0%—6/8).

### 3.5. One-Year Survial of Cardiorenal Patients

**Figure 5:**
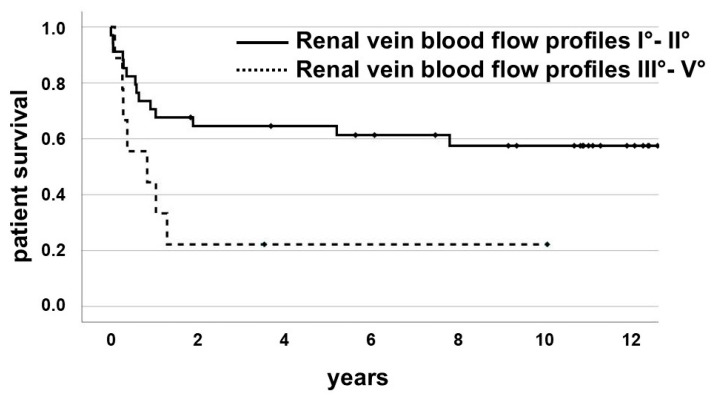
Kaplan–Meier plot of patient survival in kidney transplant patients according to the renal vein blood flow profile. Patients with graft loss due to cardiorenal syndrome showed significantly lower survival (*p* = 0.005). The 1-year patient survival rate (44.4% vs. 70.6%, log-rank: chi^2^ = 2.305, *p* = 0.129) and the cumulative patient survival rate within the follow-up (22.2% vs. 58.8%, log-rank: chi^2^ = 4.888, *p* = 0.027) were lower in patients with cardiorenal syndrome. The mean patient survival time after renal transplantation was significantly lower in the group with a higher grade of venous congestion (2.0 ± 3.2 vs. 6.3 ± 5.0 years, *p* = 0.005) than in patients with graft loss due to other reasons. There was no patient with a Grade III renal vein blood flow in this study. Patients with cardiorenal syndrome showed renal vein blood flow profiles of Grades IV and V exclusively.

Although patients with graft loss could continue to live on dialysis, the 1-year survival rate of cardiorenal patients was lower than that of patients without cardiorenal syndrome (44.4% vs. 70.6%, log-rank: chi^2^ = 2.305, *p* = 0.129), and the cumulative patient survival rate within the follow-up was significantly lower in patients with cardiorenal syndrome (22.2% vs. 58.8%, log-rank: chi^2^ = 4.888, *p* = 0.027). The mean patient survival time after renal transplantation was significantly lower in the group with a higher grade of venous congestion, defined as Grades III–V (2.0 ± 3.2 vs. 6.3 ± 5.0 years, *p* = 0.005), than in patients with graft loss due to other reasons (Table 1, Figure 5). This might be a consequence of higher cardiac comorbidity.

### 3.6. Development of Score to Predict Risk of Early Graft Failure Due to Cardiac Postrenal Venous Congestion Before Transplantation

We attempted to implement different scores to identify patients at risk of early graft loss due to postrenal venous congestion prior to transplantation (Table 5). It was possible to identify two scores with the use of multiple right heart parameters alone (Score 1) and the combination of right heart parameters with evidence of venous congestion in liver duplex sonography (Score 2).

As shown in Table 5, the first score included one point for the enlargement of the right atrium (>20 cm^2^), an enlarged right ventricular end-diastolic diameter (>36 mm), a higher pulmonary artery pressure (>33 mmHg), a reduced tricuspid annular plane systolic excursion (<16 mm), and moderate tricuspid regurgitation (one point) or severe tricuspid regurgitation (two points). Using this score, an AUC of 0.914 [0.741, 1.000] could be reached, with sensitivity/specificity of 87.5%/87.5%. The ideal cut-off, as determined by the Youden index, was >1.

Adding the presence of severe congestion in liver duplex sonography (one point) to Score 1 resulted in an AUC of 0.914 [0.741, 1.000] with sensitivity/specificity of 87.5%/91.7%. The optimal cut-off point using the Youden index was >2.

Using liver duplex sonography (with evidence of severe liver congestion) alone yielded an AUC of 0.831 [0.618, 1.000] with sensitivity/specificity of 71.4%/94.7%. The ideal cut-off using the Youden index was 1 (Table 6).

## 4. Discussion

Owing to better surgical techniques and advances in the understanding of transplant immunology, early renal graft loss within the first year is a rare event in transplant centers [4]. The reasons for early graft loss are numerous. We identified a relevant group of patients who suffered from early graft loss and showed characteristic signs of cardiorenal syndrome.

In comparison to reports from other centers [6,7,8], the rate of transplant failure due to cardiorenal syndrome is slightly higher in our transplant center (1.6% of all transplanted patients, vs. 0.56% [7] and 0.6% [8]). The proportion of cardiorenal patients among recipients with early graft failure was 20.9%. Assuming that there were more patients with recurring cardiac decompensations or later graft loss (>1 year) due to cardiorenal syndrome, the rate would probably be even higher. We also found that cardiorenal patients represented a relevant proportion within the group of recipients who died within the first year after kidney transplantation (28.6%).

To date, these cardiorenal patients have only been characterized by a combination of significantly reduced renal function together with the characteristic clinical signs of hydropic overload, some clinical evidence of heart failure, and the exclusion of other reasons for allograft dysfunction (proven by biopsy) [6,7,8]. We found typical renal vein flow profiles, which identified cardiorenal patients precisely, in contrast to all patients with early graft loss due to other reasons (Figure 3).

In contrast to other reports of cardiorenal syndrome in kidney transplant patients, we paid special attention to the duplex sonographic characteristics of the renal transplant, especially the renal vein profile. There is evidence that the renal vein flow profile is a key issue in the pathophysiology of postrenal venous congestion in cardiorenal syndrome [16]. To our knowledge, this is the first systematic study concerning the renal vein flow profile in kidney transplant patients.

We propose an adapted semi-quantitative rating scale to describe the extent of renal congestion. In our experience, continuous venous flow profiles correspond to a normal picture in the graft vein (Grade I). Grade II could be a transitional stage between the normal and pathological venous congested stages. Patients with an increasing grade of venous stasis display a discontinuous vein flow (Grades III–IV). In contrast to other rating scales [15], we added the additional aspect of venous stasis: a to-and-fro-motion within the renal vein represents significant venous backflow into the graft and is the highest degree of venous stasis (Grade V). Higher grades represent a decrease in venous flow time.

In our study, the cardiorenal group was characterized by typical discontinuous blood flow profiles within the vein of the renal transplant, indicating a high degree of venous congestion (Grades III–V). All other patients with early graft loss due to other reasons showed continuous renal vein flow profiles (Grades I and II). Grade III was not found in the patients in this study, but we have noticed this renal vein blood flow profile in patients with impending cardiorenal syndrome.

The problem with the histological confirmation of cardiorenal syndrome is that the pattern of damage seen in the renal biopsy is a non-specific pattern of severe acute tubular injury. In the initial phase of transplantation in particular, acute tubular injury is often seen as a result of the ischemia–reperfusion reaction. Therefore, a renal biopsy is primarily required to exclude other causes, such as acute rejection. It only has supplementary value in diagnosing cardiorenal syndrome.

The echocardiographic diagnosis of cardiorenal syndrome is difficult because the underlying causes are different and it could affect both the right and the left sides of the heart. In addition, the same changes did not lead to cardiorenal syndrome in the same way in all patients studied.

The duplex sonographic diagnosis of cardiorenal syndrome allows us to identify cardiorenal patients with prerenal perfusion deficit and postrenal venous congestion. Ultrasound could identify patients with increased postrenal venous congestion easily by detecting a discontinuous renal venous flow.

This work also provides approaches for the targeted treatment of the two different aspects of cardiorenal syndrome. Whereas patients with reduced prerenal perfusion need support for cardiac function, patients with postrenal venous congestion need volume restriction and volume removal in certain circumstances by dialysis.

The development of cardiorenal syndrome in end-stage renal disease patients is due to various reasons, but most patients suffer from a relevant heart disease. Risk factors for the development of cardiorenal syndrome include acute or chronic right heart failure or the presence of pulmonary hypertension [18]. The prevalence and severity of pulmonary hypertension in hemodialysis patients were much higher than those in patients who received conservative treatment for renal insufficiency and increased with the duration of dialysis therapy [19]. A possible explanation is the presence of a high-flow-volume arteriovenous fistula [19,20,21].

The influence of PAH on kidney transplant patients remains controversial. Some studies have found an association with early graft dysfunction [22], a reduced eGFR at the first two years post-transplant [23], or post-transplant patient survival [24], while others have observed no higher risk for patient or graft survival [25,26].

Besides the risk of primary non-function, the cardiorenal patients in our study showed a significantly higher risk of death within the first year after transplantation in comparison to patients with graft loss due to other reasons (55.6% vs. 29.4%, Figure 5). This raises the question of how dialysis patients who are at risk of cardiorenal syndrome could be identified in advance.

Identifying these patients might be difficult, as Waiser et al. also found that patients without any previous evidence of heart failure could also develop cardiorenal syndrome after transplantation [8]. The possibility of adjusting the volume status in dialysis patients during dialysis sessions may mask the existence of latent cardiorenal syndrome. Fluid administration, combined with simultaneous dialysis withdrawal after transplantation, revealed cardiac stress.

In our opinion, the liver Doppler profile is an easily accessible parameter that does not require any specific sonographic skills or equipment. We found, in 75% of all cardiorenal patients, a typical Doppler flow profile in the liver or the portal veins. It is noteworthy that this is not a liver duplex, which is found in patients with advanced liver disease. In these patients, a continuous monophasic blood flow would be the most common as a consequence of liver fibrosis.

The typical echocardiographic parameters are complex, and these patients did not always exhibit typical echocardiographic parameters in the pre-transplant period. Furthermore, owing to the manifold reasons for cardiac dysfunction (valvular problems, reduced left or right heart function, etc.), it is easier to obtain potentially relevant venous congestion within other organs, such as the liver. Because of the reduced size of the original kidneys in dialysis patients, and consequently the reduced blood flow, it is difficult to examine the hemodynamics of the original kidneys in dialysis patients.

A prospective study is needed to determine the significance of the portal vein flow in predicting early graft loss due to cardiac postrenal venous congestion. For this purpose, patients should be screened for the relevant portal vein flow patterns prior to kidney transplantation. Depending on the existing portal vein flow pattern, the outcome of the transplantation must be assessed.

We tried to implement a score to identify patients with a risk of early graft loss due to postrenal venous congestion. It was possible to identify two scores with the use of multiple right heart parameters alone (Score 1) and the combination of right heart parameters with evidence of venous congestion in liver duplex sonography (Score 2). The scores reached high sensitivity and specificity, but their validation was difficult due to the limited data available. The cut-off values indicated by the Youden index were relatively low and would set the threshold for the identification of patients at a relatively low level. We aim to address this problem in a further study with a larger cohort and improved data quality.

When all scores are compared in terms of complexity, the single evaluation of the liver duplex profile still seems to be promising, because this parameter could be retrieved during dialysis sessions easily and identifies patients at risk with the highest specificity of 94.7%.

If there is a predisposition to cardiorenal graft failure based on the portal vein flow or when patients are identified after kidney transplantation, there are different ways to improve the outcomes. Detecting patients prior to transplantation could prevent poor outcomes. In our opinion, assessing the portal vein flow profile is a promising and easily applicable way of identifying these patients. These patients would benefit from an intensive cardiological assessment prior to transplantation. Patients with venous congestion require euvolemia throughout the transplantation process. If possible, organs at high risk of delayed graft function should be avoided in these patients because delayed urinary production complicates volume management.

Patients with other cardiac problems (e.g., reduced ejection fraction due to coronary insufficiency) and no change in renal vein flow require improvements in their specific cardiac problems (e.g., coronary angioplasty, catecholamines).

The main limitation of this study is the retrospective approach, as it may have introduced selection bias and information bias. Although the relevant information (e.g., renal vein flow) was accessible for all patients, not all data were available for all patients (e.g., portal vein flow prior to transplantation). This could affect the reliability of the findings. With only 43 patients experiencing early graft loss and just nine cases attributed to cardiorenal syndrome, the sample size, particularly for the key subgroup, was very small. This limits the statistical power and the generalizability of the results, especially given the rarity of the condition studied. To enlarge the number of cardiorenal patients, a future study with a multicentric design is needed, especially because graft loss due to venocongestive patients is a rare finding.

During the course of the study, we identified liver duplex sonography as a key diagnostic tool to identify patients at risk for cardiorenal syndrome; this is implemented in our pre-transplant work-up and gives the opportunity to examine this issue in a standardized manner.

## 5. Conclusions

In conclusion, in our study, cardiorenal syndrome was the reason for early graft loss in 22% of patients with early graft failure. We identified typical renal vein blood flow profiles to identify patients with cardiorenal syndrome following a renal transplant, especially in patients with increased postrenal venous congestion. According to our analysis, we were able to find a potential predictive parameter in the liver blood flow profile before transplantation to identify patients at risk for cardiorenal syndrome. Although these retrospective findings must be proven in further studies, we hope that our work will help to improve the outcomes of renal transplantation.

## Figures and Tables

**Table 1:** Characteristics of patients with early graft failure and organ quality.

	All Patients	Patients with Other Reasons for Graft Loss (N = 34)	Patients with Assumed Cardiorenal Syndrome (N = 9)	*p*-Value
Recipient age	57 ± 12	56 ± 12	61 ± 10	0.276
Time on dialysis (mo.)	37 ± 31	39 ± 31	27 ± 28	0.332
Donor age	55 ± 17	54 ± 17	59 ± 16	0.419
Multi-organ recipient	7	5	2	
Heart transplantation	3	1	2	
HLA mismatches (cumulative for A, B, DR)	3.6 ± 1.6	3.5 ± 1.8	4.1 ± 0.7	0.347
PRA (%)	6 ± 16	6 ± 16	7 ± 14	0.812
Living donation	3	3	0	
Cold ischemia time (hours)	11.7 ± 4.7	11.6 ± 4.9	12.2 ± 3.3	0.775
Warm ischemia time (min.)	44 ± 18	43 ± 19	48 ± 16	0.581
Histological evidence of rejection (including borderline rejection)	12/35 (34%) (NA 7)	10/27 (37%) (NA 6)	2/8 (25%) (NA 1)	
Histological evidence of singular acute tubular necrosis	11/35 (31%) (NA 7)	8/27 (29%) (NA 6)	3/8 (37%) (NA 1)	
One-year survival rate	28/43 (65.1%)	24/34 (70.6%)	4/9 (44.4%)	0.129
Overall survival rate	22/43 (51.2%)	20/34 (58.8%)	2/9 (22.2%)	0.027
Patient survival (years)	5.4 ± 4.9	6.3 ± 5.0	2.0 ± 3.2	0.005
Death age	59 ± 11	59 ± 11	60 ± 11	0.985

**Table 2:** Cardiac parameters before kidney transplantation.

	All Patients (N = 43)	Patients with Other Reasons for Graft Loss (N = 34)	Patients with Assumed Cardiorenal Syndrome (N = 9)	*p*-Value (Pearson’s Chi^2^, Student’s *t*-Test)
**Cardiac disease**				
Any heart disease (myocardial, valvular, and/or coronary heart disease, %)	80.0 (20/34)	46.2 (12/26)	100 (8/8)	*p* = 0.07, φ = 0.46
Any valvular heart disease (%)	36.4 (12/33)	20.0 (5/25)	87.5 (7/8)	*p* ≤ 0.001, φ = 0.60
Coronary artery disease (%)	28.6 (12/42)	23.5 (8/34)	50.0 (4/8)	*p* = 0.136, φ = 0.23
Atrial fibrillation (%)	4.8 (2/42)	2.9 (1/34)	12.5 (1/8)	*p* = 0.253, φ = 0.18
**Right heart**				
Any right heart disease (%)	50.0 (15/30)	36.4 (8/22)	87.5 (7/8)	*p* = 0.01, φ = 0.45
Right atrial area dilatation (>20 cm^2^)	48.0 (12/25)	36.8 (7/19)	83.3 (5/6)	*p* ≤ 0.05, φ = 0.40
RVEDD enlarged (>36 mm)	28.6 (8/28)	9.5 (2/21)	85.7 (6/7)	*p* ≤ 0.001, φ = 0.73
TAPSE reduced (<16 mm)	14.3 (4/28)	0.0 (0/22)	66.7 (4/6)	*p* ≤ 0.001, φ = 0.78
TAPSE (mm)	17 ± 3	20.5 ± 0.5	16 ± 3	n/a
Tricuspid regurgitation - missing/mild (%) - moderate (%) - severe (%)	75.9 (22/29) 10.3 (3/29) 13.8 (4/29)	90.5 (19/21) 4.8 (1/21) 4.8 (1/21)	37.5 (3/8) 25.0 (2/8) 37.5 (3/8)	*p* ≤ 0.001, φ = 0.56
sPAP elevated (>33 mmHg; %)	37.5 (9/24)	18.6 (3/16)	75.0 (6/8)	*p* = 0.005, φ = 0.56
Mean pulmonary artery pressure (mmHg)	33 ± 14 (n/a 19)	28 ± 9 (n/a 18)	44 ± 18 (n/a 1)	0.015
**Left heart**				
Any left heart disease (%)	80.0 (20/34)	46.2 (12/26)	100 (8/8)	*p* = 0.01, φ = 0.44
Left atrial area (>20 cm^2^; %)	52.9 (18/34)	38.5 (10/26)	100 (8/8)	*p* = 0.002, φ = 0.52
Left ventricular end-diastolic diameter (LVEDD) enlargement (F > 52 mm; M > 58 mm; %)	28.6 (6/21)	21.4 (3/14)	42.8 (3/7)	*p* = 0.31, φ = 0.22
LVEF (%) - Normal (>52%) - Mild (41–51%) - Moderate (30–40%) - Severe (<30%)	80.0 (28/35) 8.6 (3/35) 5.7 (2/35) 5.7 (2/35)	85.2 (23/27) 11.1 (3/27) 3.7 (1/27) 0.0 (0/27)	62.8 (5/8) 0.0 (0/8) 12.5 (1/8) 25.0 (2/8)	*p* = 0.03, φ = 0.50
Evidence of diastolic dysfunction (%)	29.0 (9/31)	19.2 (5/26)	80.0 (4/5)	*p* = 0.006, φ = 0.49
**Liver duplex sonography**				
Evidence of venous congestion in liver duplex sonography (%)	22.9 (8/35)	7.4 (2/27)	75.0 (6/8)	*p* ≤ 0.001, φ = 0.68

**Table 3:** Sonographic parameters of kidney transplant patients.

	All Patients (N = 43)	Patients with Other Reasons for Graft Loss (N = 34)	Patients with Assumed Cardiorenal syndrome (N = 9)	*p*-Value
Renal length (cm)	10.4 ± 1.1	10.6 ± 1.1	10.1 ± 1.1	0.281
Resistive Index (RI)	0.73 ± 0.09	0.72 ± 0.09	0.75 ± 0.1	0.397
Renal vein profile				
- Grade I - Grade II - Grade III - Grade IV - Grade V	33 1 0 3 6	33 1 0 0 0	0 0 0 3 6	

**Table 4:** Clinical and sonographic features used to identify cardiorenal patients.

	Unexplained Renal Insufficiency	Severe Heart Disease	Severe Volume Overload	Acute Tubular Necrosis	Combination of All	Renal Venous Congestion Grades IV and V
Patient 1	Yes	Yes	Yes	Yes	Yes	Yes (V)
Patient 2	Yes	Yes	Yes	No	No	Yes (V)
Patient 3	Yes	Yes	Yes	N/a	Yes	Yes (V)
Patient 4	Yes	Yes	Yes	No	No	Yes (IV)
Patient 5	Yes	Yes	Yes	Yes	Yes	Yes (V)
Patient 6	Yes	Yes	Yes	No	No	Yes (V)
Patient 7	Yes	Yes	Yes	No	No	Yes (V)
Patient 8	No (rejection)	Yes	Yes	No	No	Yes (IV)
Patient 9	Yes	Yes	Yes	Yes	Yes	Yes (IV)

**Table 5:** Scoring system to predict the probability of graft loss due to venous congestion.

**Score 2**	**Score 1**	**Criterion**	**Points**
		RA area > 20 cm^2^	1
		RVEDD > 36 mm	1
		PAP > 33 mmHg	1
		TAPSE < 16 mm	1
		Tricuspid regurgitation II°	1
		Tricuspid regurgitation III°	2
	**+**	Liver duplex sonography with evidence of venous congestion	1

**Table 6:** ROC analysis of the different scores to predict the probability of graft loss due to venous congestion.

Score	AUC	CI	Sensitivity	Specificity	Cut-Off
Score 1	0.914	0.741, 1.000	87.5%	87.5%	>1
Score 2	0.914	0.741, 1.000	87.5%	91.7%	>2
Liver duplex sonography with evidence of venous congestion	0.831	0.618, 1.000	71.4%	94.7%	1

## Data Availability

The data that support the findings of this study are available upon request from the corresponding author. The data are not publicly available because of privacy concerns and the data policy of the institution.

## Abbreviations

The following abbreviations are used in this manuscript:

A.	Artery
Aa.	Arteries
F	Female
HLA	Human leucocyte antigen
LVEDD	Left ventricular end-diastolic diameter
LVEF	Left ventricular ejection fraction
M	Male
mo.	Months
n/a	Not applicable
PAH	Pulmonary arterial hypertension
PRA	Panel-reactive antibodies
RI	Resistive index
RVEDD	Right ventricular end-diastolic diameter
sPAP	Systolic pulmonary artery pressure
TAPSE	Tricuspid annular plane systolic excursion
UTI	Urinary tract infection
V.	Vein
Vv.	Veins
